# Age-dependent changes in anti-Müllerian hormone levels in Lebanese females: correlation with basal FSH and LH levels and LH/FSH ratio: a cross-sectional study

**DOI:** 10.1186/s12905-020-00998-4

**Published:** 2020-06-26

**Authors:** Eddie Racoubian, Gulzhanat Aimagambetova, Ramzi R. Finan, Wassim Y. Almawi

**Affiliations:** 1 St. Marc Medical and Diagnostic Center, Ashrafieh, Beirut, Lebanon; 2 School of Medicine, Nazarbayev University, Nur-Sultan, Astana Kazakhstan; 3 Department of Obstetrics and Gynecology, Hôtel-Dieu de France, Beirut, Lebanon; 4 Faculte’ des Sciences de Tunis, Universite’ de Tunis El Manar, Tunis, Tunisia; 5 College of Health Sciences, Abu Dhabi University, Abu Dhabi, United Arab Emirates

**Keywords:** Anti-Müllerian hormone, Follicle-stimulating hormone, Luteinizing hormone, Menopause

## Abstract

**Background:**

To investigate the age-dependent changes in circulating anti-Müllerian hormone (AMH) levels in healthy Arabic-speaking Lebanese women, and to correlate changes in serum AMH levels with serum FSH and LH values, and LH/FSH ratio.

**Methods:**

Cross-sectional study, involving 1190 healthy females, age 17–54 years, with regular menses and both ovaries. Serum AMH levels (ng/ml) were measured by ELISA.

**Results:**

There was an inverse proportion of AMH and subject’s age, which declined from median 6.71 (2.91) ng/ml in young subjects, to 0.68 (0.45) ng/ml in subjects older than 50 years. Average yearly decrease in median AMH levels was 0.27 ng/ml/year through age 35, but then diminished to 0.12 ng/ml/year afterwards. Receiver operating characteristic curve analysis demonstrated high sensitivity and specificity of age as determinant of AMH levels. In contrast to AMH, FSH levels increased progressively from 5.89 (0.11–62.10) ng/ml in young subjects, to 38.43 (3.99–88.30) ng/ml in subjects older than 50 years. On the other hand, age-dependent changes in LH/FSH ratio paralleled those of AMH. Linear regression modeling testing the independent effect of AMH on FSH and LH, adjusted for age, showed that AMH was significant predictor of FSH and LH/FSH ratio, but not LH. This did not contribute significantly to baseline LH and FSH prediction.

**Conclusions:**

Circulating AMH levels are inversely related to age as also shown elsewhere, and are predictors of LH/FSH ratio and FSH but not LH levels in eumenorrheic females.

## Background

Anti-Müllerian hormone (AMH) is 140 kDa disulfide-linked homodimeric glycoprotein, belonging to transforming growth factor-β (TGF-β) superfamily [1], is essential factor involved in the regression of Müllerian ducts in the male fetus (reviewed in Josso [2]). AMH is produced in high amounts by Sertoli cells in males from testicular differentiation up to puberty, and in lower amounts by granulosa cells of primary and small antral follicles in females from the second half of intrauterine life up to menopause [3, 4]. The main role of AMH is regulation of fetal male sex differentiation, while other roles in ovarian follicular differentiation and elsewhere have been described (reviewed in Josso [2]).

AMH is activated by proteolytic cleavage of pro-protein, and binds specific AMH type 2 receptor [4], followed by the recruitment of SMAD signal transducer proteins [5], leading to their nuclear translocation where they regulate target gene expression [1, 4, 5]. Along with its role as determinant of the male sexual differentiation, changes in AMH levels, together with follicle-stimulating hormone (FSH) and luteinizing hormone (LH), reflect aging in females [6]. This age-dependent decline in fertility typically begins at the third decade of female’s life and deteriorates markedly after age 35 years old. This decline is attributed to gradual age-related decrease in the pool of ovarian follicles [7], coupled with increases in follicular-phase serum FSH and LH levels [8].

While LH and FSH levels are determinants of ovarian activity, AMH levels reflect ovarian reserve. AMH serum levels are reliable indicators of ovarian reserve (follicular pool) in reproductive age women [3, 9], as they remain constant throughout the menstrual cycle [10, 11], with low variability in subsequent cycles [2], and are not affected by endocrine perturbations [5, 10, 11]. Clinical studies demonstrated that decreased AMH levels indicates reduced ovarian responsiveness to exogenous gonadotropin administration, and poor pregnancy outcome in women undergoing infertility treatment [9]. Serum AMH levels correlate with follicle count [12, 13], and are more accurate than age and other conventional markers (FSH, estradiol, inhibin B) in predicting pre-ovulatory oocyte supply in response to ovulation induction [14]. Clinically, AMH determination is utilized in assessing ovarian reserve in infertility diagnosis, premature ovarian failure, and polycystic ovary syndrome (PCOS) [2, 15]. A recently published systematic review reported that AMH was the most promising predictive markers for ovarian aging, and timing of menopause [16].

Unlike other reproductive hormones, AMH is detectable in females of all ages. Circulating AMH levels show only minor fluctuations during childhood and adolescence [17]. The negative AMH-FSH correlation in prepubertal girls supports the notion that AMH is a quantitative marker of ovarian follicles even in young girls [17]. In adults, AMH levels peak in the early twenties [18], but begin to decline after that [19], and are virtually undetectable during menopause [19]. However, the timing of menopause appears to vary according to the ethnic/racial background, exemplified by the early onset of menopause in Africans and delayed onset of menopause in Asians [20]. Few multi-ethnic studies confirmed decline in AMH as determinant of menopause [21, 22], suggesting race/ethnic contribution to differences in ovarian reserve and timing of menopause, and variation in risk for post menopause-associated disease [21].

Given the progressive rise in the need for in vitro fertilization (IVF) in Lebanon, which often requires multiple ovarian stimulation/embryo transfer cycles due to many factors, including age, type of infertility treatment, and AMH basal levels [23], we investigated the age-dependent changes in circulating AMH levels in a large group of healthy Lebanese women. In addition, we report on the contribution of altered AMH levels on FSH and LH values.

## Methods

### Study subjects

The cross-sectional study was performed at St. Marc Medical Center, an integrated clinical diagnostics center located in East Beirut. Between 2010 and 2015, 1190 healthy volunteer women, age 17–54 years, were recruited, after obtaining information on age and area of residence. Inclusion criteria were regular menses (duration of cycle: 25–35 days, with 5 days or less inter-cycle difference), and presence of both ovaries. Exclusion criteria included current or hormone therapy in the past 6 months, history of confirmed infertility, PCOS, overt autoimmune disease, along with chronic, metabolic, and endocrine disease (including hyperandrogenism). After disclosing information about study subjects’ reproductive history and regularity of their menstrual cycle, written informed consent were obtained from them followed by peripheral venous blood samples for AMH, FSH and LH levels assessment. All blood samples were collected on day 3 of the same menstrual cycle. St. Marc Medical Center Research and Ethics Committee (SMMC-RE02–01/09; granted on 7 March 2009) approved the study protocol, which was done according to Helsinki II guidelines.

### AMH assay

Blood samples for AMH determination were collected in plain tubes, allowed to clot for 15–20 min, and were centrifuged at 4000 rpm for 10 min, and serum aliquots were stored at − 20 °C; freeze-thawing was avoided. Serum AMH was measured by AMH Gen II ELISA kit (Beckman Coulter, Brea, CA). AMH concentrations were expressed in ng/ml (conversion factor: 1 ng/ml = 7.14 pM). The assay detection limit was 0.14 ng/ml; intra- and inter-assay coefficients of variation were 5–9% and 7–12%, respectively. Samples which were below the limit of detection of AMH (< 2 pg/mL), were excluded to avoid using null values. FSH (mIU/ml) and LH (mIU/ml) were quantitated using Cobas e411 (Roche Diagnostics, Indianapolis, IN).

### Statistical analysis

AMH levels were presented as mean ± SD, and the 5th, 25th, 50th, 75th, and 95th percentiles were determined with SPSS v. 23 (IBM, Armonk, NY). Study subjects were stratified into eight age categories: 17–20 years, 20–25 years, >25–30 years, >30–35 years, >35–40 years, >40–45 years, >45–50 years, and 51–61 years. Differences between age, AMH, FSH, LH, and LH/FSH ratio between the eight groups were determined by ANOVA; *P* < 0.05 considered statistically significant. Multivariate hierarchical linear regression modeling was performed to assess the independent effect of AMH on LH, FSH, and LH/FSH ratio, after adjusting for age as the independent variable. Beta coefficients (SE) and 95% confidence intervals (CI), as well as *P* values were reported for these models.

## Results

### Age-dependent decline in AMH levels

**Fig. 1:**
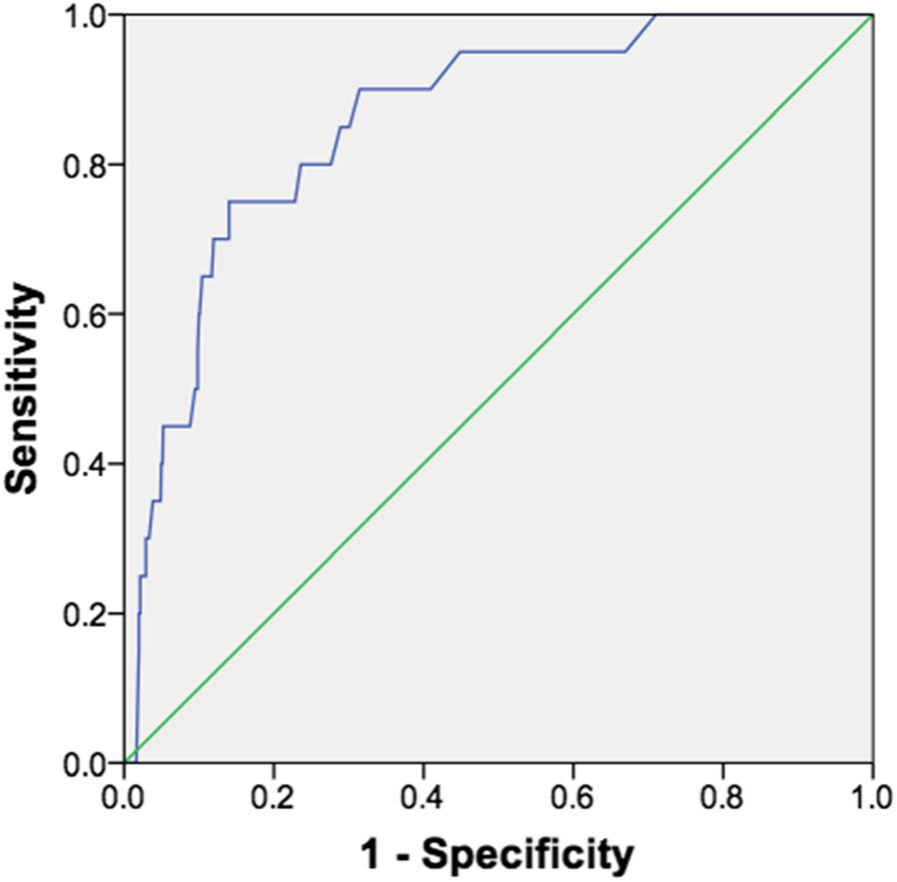
ROC curve of serum levels of AMH changes according to age. The Spearman’s correlation coefficient between AMH and age was − 0.339 (*P* < 0.0001), and the area under ROC curves of AMH was 0.857 ± 0.038 (asymptomatic 95% CI = 0.783–0.932)

Table 1 summarizes the mean and median AMH values among 1190 female participants, who were grouped into eight age groups. At blood sampling, there were 27 women younger than 20 years, and 13 women older than 50 years of age at blood sampling; most (879; 73.9%) were in the 30–45 year age categories. The mean (± SD) and median AMH values recorded for unselected study participants were 2.47 ± 2.29 and 1.80, respectively. There was an inverse proportion of AMH and subject’s age (*P* < 0.001), which declined from 5.14 ± 3.21 ng/ml in the 20–25 year age group, to 0.68 ± 0.45 ng/ml in women older than 50 years (Table 1). The average yearly decrease in median AMH levels was 0.27 ng/ml/year through age 35, but then diminished to 0.12 ng/ml/year after age 35 (Table 1). AMH 5th, 25th, 50th, 75th, and 95th percentile analysis confirmed the age-decline in AMH levels (Table 1). Receiver operating characteristic (ROC) curve analysis demonstrated high sensitivity and specificity of age as determinant of AMH levels, and Spearman correlation coefficient value obtained was − 0.339, and largest area under the curve (0.857 ± 0.038; 95%CI = 95.6–100) was obtained (Fig. 1).

**Table 1:** Age-specific AMH levels for 1190 Lebanese women at defined age intervals

AMH Perecntiles ^I^									
Age groups	Number	Menopausal	Median	Mean ± SD	5th	25th	50th	75th	95th
ALL	1190	36 (3.0) ^2^	1.80	2.47 ± 2.29	0.45	0.85	1.80	3.10	7.44
17–20 years	27	0 (0.0)	7.25	6.71 ± 2.91	1.40	4.40	7.25	9.50	10.80
20–25 years	55	0 (0.0)	4.47	5.14 ± 3.21	0.93	2.80	4.47	7.00	12.04
> 25–30 years	111	0 (0.0)	3.60	4.54 ± 2.90	1.00	2.40	3.60	6.50	11.00
> 30–35 years	219	0 (0.0)	2.55	3.19 ± 2.11	0.95	1.95	2.55	3.95	7.20
> 35–40 years	306	0 (0.0)	1.80	2.23 ± 1.75	0.51	1.00	1.80	2.80	6.05
> 40–45 years	354	5 (1.4)	1.00	1.37 ± 1.01	0.42	0.70	1.00	1.75	3.61
> 45–50 years	105	19 (18.1)	0.70	0.89 ± 0.63	0.27	0.50	0.70	1.00	2.38
51–61 years	13	12 (92.3)	0.60	0.68 ± 0.45	0.20	0.43	0.60	0.70	0.94

^1^*P* < 0.001 among different groups

^2^Number of subjects (percent total within group)

### Age-dependent changes in FSH and LH relative to AMH

In contrast to AMH, FSH follow the opposite direction. FSH values progressively increased from [median (range)] 5.89 (0.11–62.10) ng/ml in the 20–25-year category females to 9.17 (0.18–167.00) ng/ml in 40–45 year-old women, and further after the age 50 years. No clear trend for age-related changes in LH levels were seen (*P* = 0.299). On the other hand, age-dependent changes in LH/FSH ratio paralleled those of AMH; they dropped from 1.16 (0.11–7.59) in 20–25 year-old females to 0.56 (0.27–2.78) in 45–50 year-old females (Table 2).

**Table 2:** Comparison of age, AMH, LH, FSH and LH/FSH ratio in different age groups

Age groups	N	Age	AMH (ng/ml)	FSH	LH	LH/FSH ratio
ALL	1191	37.63 ± 7.20	2.47 ± 2.29	7.96 (0.09–177.80)	7.02 (0.10–113.60)	0.72 (0.02–7.59)
17–20 years	27	19.04 ± 2.17	6.71 ± 2.91	5.56 (4.46–60.61)	13.62 (2.08–68.42)	1.63 (0.47–5.08)
20–25 years	55	23.46 ± 1.27	5.14 ± 3.21	5.89 (0.11–62.10)	7.43 (0.10–35.82)	1.16 (0.11–7.59)
> 25–30 years	111	28.20 ± 1.47	4.54 ± 2.90	6.06 (0.78–23.91)	6.51 (0.10–13.71)	1.03 (0.13–2.13)
> 30–35 years	219	33.15 ± 1.39	3.19 ± 2.11	7.44 (3.29–177.80)	6.42 (1.48–84.80)	0.78 (0.19–4.47)
> 35–40 years	306	38.28 ± 1.44	2.23 ± 1.75	7.38 (0.09–160.30)	7.22 (0.10–75.71)	0.77 (0.02–3.84)
> 40–45 years	355	43.01 ± 1.43	1.37 ± 1.01	9.17 (0.18–167.00)	6.84 (0.10–113.60)	0.64 (0.18–4.00)
> 45–50 years	105	47.35 ± 1.35	0.89 ± 0.63	11.31 (2.30–108.40)	7.77 (3.39–50.72)	0.56 (0.27–2.78)
51–61 years	13	53.15 ± 2.79	0.68 ± 0.45	38.43 (3.99–88.30)	24.10 (2.07–51.93)	0.58 (0.18–0.75)
*P*			< 0.001	1.09 × 10^−7^	0.942	4.42 × 10^−4^
Chi square			86.88	45.51	2.29	26.32

### Correlation between AMH and LH/FSH levels

Multivariate hierarchical linear regression modeling was developed to assess the independent effect of AMH on FSH and LH, adjusted for age. Beta coefficients (SE) and *P* values were reported for these models. Results from Table 3 indicated that AMH was a significant predictor of FSH (*P* = 0.029), but not LH (*P* = 0.568), along with LH/FSH ratio (*P* < 0.001). This persisted after controlling for age, which did not contribute significantly to baseline LH and FSH prediction.

**Table 3:** Regression analysis of AMH levels as predictors of LH and FSH levels

	Unadjusted			Age-Adjusted		
	***P***	β (SD)	95% CI	***P***	β (SD)	95% CI
LH	0.065	- 0.042 (0.023)	−0.086 — 0.003	0.568	0.006 (0.010)	−0.014 — 0.025
FSH	0.729	−0.005 (0.014)	−0.032 — 0.032	0.029	−0.035 (0.016)	− 0.066 — -0.004
LH/FSH	1.95 × 10^− 8^	1.369 (0.238)	0.901–1.837	7.62 × 10^− 10^	1.081 (0.170)	0.746–1.416

## Discussion

This study was prompted by the need for reliable marker of diminishing ovarian function, apart from FSH and estradiol [18;24], and independent of the phases of the menstrual cycle [10, 19]. In view of its utility in evaluating fertility (*ovarian reserve*), assessment of age-specific variation in AMH levels is central for infertility workup [9, 12], as serum AMH reflects AMH production only from functioning follicles [24]. This is the first study that addresses age-specific serum AMH levels in 1190 Arabic-speaking Lebanese women, and spans the reproductive lifespan from 17 to 54 years.

The inverse relationship between serum AMH and age was previously reported for several ethnic groups, and our findings on Lebanese women confirm this negative association. The kinetics of AMH decline was paralleled with a similar decline in LH/FSH ratio, both of which were inversely related to FSH or LH levels but was attenuated for FSH was when validated by regression analysis. Insofar as the timing of natural menopause, and age-dependent reduction in AMH vary according to race and ethnicity [20, 21], this study identifies population-based reference range for AMH concentration and yearly decline levels in Lebanese women.

In agreement with earlier findings, marked heterogeneity in AMH values were seen among our cohort of Arabic-speaking Lebanese women, especially among younger compared with older women, suggesting a role in follicular development [19, 25, 26]. The impact of age on decline of AMH levels was analyzed at two levels, as continuous and later categorical (5-year age groups), and confirmed by ROC analysis (area under ROC curve = 0.857). This was in agreement with earlier studies demonstrating that ageing is linked with altered AMH expression, irrespective of follicular cohort [9], and that patients with advancing age (hence low follicular count) had drastically low levels of AMH when compared to patients with a higher follicular count [10, 26]. The AMH percentiles (5th, 25th, 50th, 75th and 95th) obtained in our study was reminiscent of the age-related normograms (5th, 25th, 50th, 75th, and 95th AMH percentiles) reported earlier [27].

AMH and FSH are highly correlated [19], and age-specific fluctuations in their values was previously demonstrated [25]. While the mean (and median) FSH and LH established for Lebanese women are consistent with those reported for healthy women in other ethnic groups, the wide variation in FSH (*n* = 30) and LH (*n* = 10) suggest the presence of an unidentified conditions. The selection of the study subjects relied on self-reported health condition, and thus the abnormally high values of FSH and LH seen in 30 and 10 participants, respectively, can be explained by undiagnosed or asymptomatic condition (including PCOS) [28], contribution of modifying factors (especially smoking) [29], status (and days) of the menstrual cycle, and varied assay conditions.

On the other hand, AMH determination at any day of a normal menstrual cycle was shown to be predictive of baseline FSH and LH levels [3, 10, 11]. Similar to AMH, FSH/LH ratio reflects ovarian reserve and is used as a laboratory predictor of diminished ovarian reserve and forecaster of response to controlled ovarian stimulation [30]. The benefit of the FSH/LH ratio is that it uses already standardized and universally obtained day 3 laboratory values [30]. This was also shown for normo-ovulatory [13], but not women with PCOS [31].

AMH determination was proposed as predictor of menopause [2, 16, 32], and very low, even undetectable, AMH levels are commonly seen five years prior to menopause. By comparison, the predictive value of FSH levels as determinant of aging predictor is lower than that of AMH, since AMH levels decline earlier than FSH [16, 32]. Our findings are consistent with the physiologic changes associated with aging in females [5]. Our results showed AMH levels were high predictors of LH/FSH ratio, more so than FSH levels, while LH levels did not correlate with AMH levels. Mixed association of FSH and AMH levels were reported [13, 26], which are likely attributed to ethnic variation [20–22], and presence of comorbidities [24, 27, 31, 33]. A significant negative correlation was found between LH/FSH ratio and age, which paralleled that of AMH, in agreement with a recent study [13]. This suggests that LH/FSH ratio is surrogate for AMH level in situations and centers where AMH measurement may not be feasible, as suggested [13].

## Conclusions

In conclusion, our study confirms the age-specific changes in AMH levels, along with LH/FSH ratio, which in turn translates into a reliable way of determining ovarian reserve, more so than FSH or LH. This does not indicate a direct feedback mechanism between AMH and LH or FSH. Instead, we favor the notion that they are independent indicators of ovarian reserve. Strengths of this study include the availability of AMH data of females aged 17–54 years, thus allowing modeling of age dependent AMH profile. In addition, the concurrent measurement of LH and FSH with AMH are best suited to study the association between AMH and both hormones and their ratio, and that given the profile of participating women, results obtained are likely representative of general female population. Our study had some shortcomings as well. Our study comprised only healthy females, thus questioning the generalizability of the findings on women with infertility and metabolic abnormalities, including PCOS [15, 24, 31] and infertility [12, 14, 19], and vitamin D deficiency [34]. Furthermore, our study involved Lebanese women, thus necessitating parallel investigations on women from related and distant ethnic backgrounds. Despite these shortcomings, our results confirm the superiority of AMH determination in the follow up of ovarian reserve, given the stability of AMH throughout the cycle and ease of sampling during the day.

## Data Availability

The datasets used and/or analyzed during the current study are available from the corresponding author on reasonable request.

## Abbreviations

AMH: Anti-Müllerian hormone
CI: Confidence intervals
FSH: Follicle-stimulating hormone
GnRH: Gonadotropin-releasing hormone
IVF: in vitro fertilization
LH: Luteinizing hormone
PCOS: Polycystic ovary syndrome
ROC: Receiver operating characteristic
SD: Standard deviation
SE: Standard error
